# Molecular diagnosis of *Taenia saginata* from two patients in Palestine: two case reports

**DOI:** 10.1186/s13256-024-04351-3

**Published:** 2024-02-14

**Authors:** Mohammad Asees, Issam Jawabreh, Omar Hamarsheh, Ziad Abdeen, Ayoub Assi, Kifaya Azmi

**Affiliations:** 1 Hematology Department, Professional Labs, Ramallah, Palestine; 2 Routine and Body Fluids Department, Professional Labs, Ramallah, Palestine; 3 Department of Life Sciences, Al-Quds University, Jerusalem, Palestine; 4 Al-Quds Public Health Society, Jerusalem, Palestine; 5 Faculty of Medicine, Department of Biochemistry, Al-Quds University, Jerusalem, Palestine

**Keywords:** *Taenia saginata*, *COX-1*, Palestine, Molecular diagnosis

## Abstract

**Background:**

Taeniasis, is a worldwide foodborne zoonotic disease caused by two principal species; *Taenia saginata* and *Taenia solium.* The tapeworm infects the intestine causing taeniasis in humans. Taeniasis is a very rare parasitic infection in Palestine with very few annual cases of unknown species. The infection rate and the disease status are not clear due to the lack of reports about the actual number of patients.

**Case presentation:**

Two Palestinian patients; one male of 22 years old from Hebron and the other is female of 33 years old from Ramallah were referred to Palestinian Health Services in the West Bank, Palestine, complained of weight loss, abdominal pain and presence of motile segments of creamy color in the their stool. Microscopic analysis of the stool samples from infected cases revealed *Taenia* eggs and proglottids, confirmed taeniasis infection. The parasite species was identified as *T. saginata* by polymerase chain reaction (PCR) amplification and sequencing of the cytochrome oxidase -1 (*COX*-1) gene.

**Conclusion:**

Taeniasis is an unusual parasitic infection in Palestine, there is a growing concern that the actual numbers of infected individuals are much higher and the occurrence of human taeniasis is principally due to people’s eating habits in consumption of raw or undercooked beef meat. This report highlighted for the first time the existence of taeniasis infection in the country; which necessitates the need to conduct further research and surveillance to reveal the actual infection rate and the available *Taenia* species.

## Background

Human taeniasis is a parasitic and zoonosis infection caused by helminths; genus *Taenia* (Linneus 1758) (Cyclophyllidea, Taeniidae). *Taenia saginata*, *Taenia solium*, and *Taenia asiatica* are the causative agents of human taeniasis. The taenia infection caused by *Taenia saginata* and *Taenia solium* distributed globally, while *T. asiatica* is principally distributed in Asian countries [1–3]. *T. saginata*; a significant cestode with a worldwide distribution. The adult tapeworm develops in the human intestine where thousands of eggs excreted and discharged in feces [4]. Patients infected with taeniasis have a broad symptoms, including but not limited to abdominal pain, weight loss, nausea, and fever [4].The most and principal and distinctive sign is the discharge of *Taenia* proglottids in the stool. Routine microscopic examination of stool samples of suspected patients may reveal the presence of *Taenia* ova. *T. saginata* life cycle include humans as a definitive host. In Palestine, taeniasis infection is very rare [5].

## Cases presentation

**Fig. 1:**
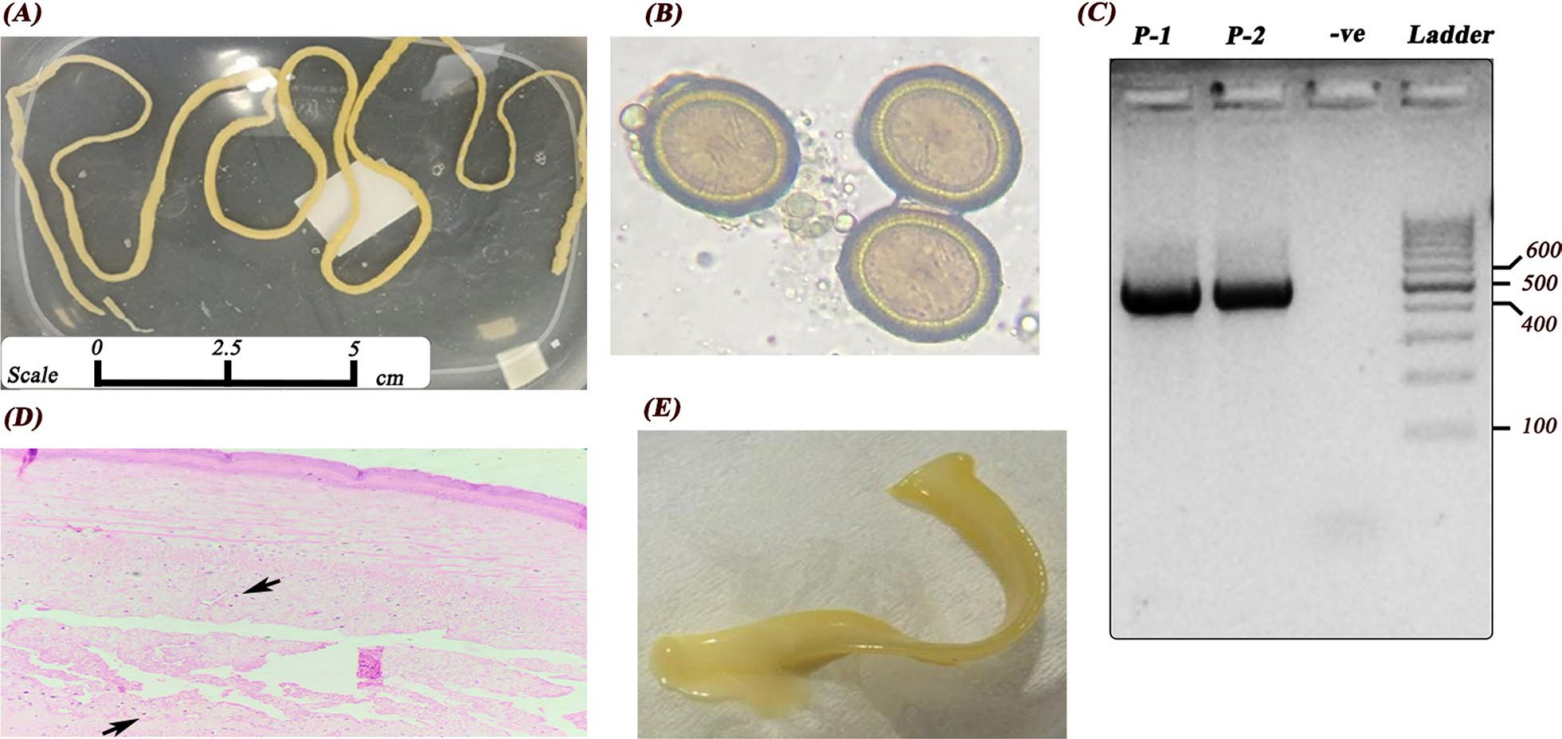
*Taenia saginata* tapeworm diagnosed from Palestinian patients. **A** A complete tapeworm strobila of *T. saginata* discharged out of the body and isolated from the stool sample of a 22 year old male patient 2 (P-2). **B** *Taenia* ova diagnosed after examination of stool samples in wet smear preparations (100 × magnification). **C** Amplification of the *COX-1* mitochondrial gene (band size 491 bp); P-1 and P-2 are amplified samples from patient 1 and patient 2, respectively, − ve is a negative control and Ladder is 100 bp DNA size marker. **D** Cross-section of a proglottid discharged from a patient and picked up from his stool sample, stained with hematoxylin and eosin and shows the thick outer tegument and the loose parenchyma filling the body with numerous calcareous corpuscles (black arrows). **E** *Taenia* proglottids discharged in stool samples of patients

Two patients were referred to Palestinian health services in the West Bank; the first one is a 33-year-old married female from Ramallah, house wife and mother of three children; presented to Ramallah medical center complaining of weight loss, transient abdominal pain, and vomiting during the night. The patient referred to gynecology clinic; clinical examination of the patient showed no physical or neurological illness. The patient reported that she is not pregnant and never smoke or drink any alcoholic beverages or complaining of any health problems necessitates taking medications prior to this case. The patient also reported the presence of proglottids segments of creamy color, motile, and abut 2–5 cm in length. As the discharge of proglottids segments continued and increased, the patients were referred to the parasitology laboratory, where a stool sample from patient has been analyzed. The microscopic stool examination confirmed the presence of the *Tenia* segments as well as *Taenia* ova (Fig. 1). Biochemistry and immunology tests of blood sample were normal; moderate increase of eosinophils count (1200), and IgE level is normal. Patient reported, she often tasted ground raw beef meat from time to time.

The second patient is a 22-year-old man from Hebron, not married, works as butcher, smoker and nonalcoholic. The patient did not report of the use of any drugs and he did not complain of any prior illness before this case. The patient referred to Hebron Hospital, Hebron, Palestine, his medical report showed that he was diagnosed with taeniasis about 5 years ago. The patient complained with weight loss, abdominal pain, general fatigue, and recurrent episodes of taeniasis infection (2–5 months). The patient reported, he has a history of eating raw beef meat and raw kidneys from cattle. The stool analysis showed proglottids segments discharged and appeared clearly in his stool sample. The biochemistry and immunology tests of his blood were normal; normal IgE level and eosinophil count respectively.

The parasitological findings after microscopic examination of a stool sample include presence of proglottids and *Taenia* ova and PCR amplification is shown in Fig. 1. None of the patients reported any travel abroad in the past six months of the confirmed diagnosis.

Based on the results of stool examination, both patients were referred to the gastroenterology clinics in Ramallah and Hebron, respectively where they were diagnosed with taeniasis and offered Albendazole (400 mg) as anti-helminthic treatment.

To identify *Taenia* species; DNA were extracted from proglottids samples discharged from both patients using Qiagen DNA extraction kit (Qiagen, Germany) following the manufacturer’s instructions, then the DNA samples were amplified using the mitochondrial cytochrome c oxidase 1 (*COX-1*) gene. The PCR amplification was performed with primers T1F (5′-ATA TTT ACT TTA GAT CAT AAG CGG-3′) and T1R (5′-ACG AGA AAA TAT ATT AGT CAT AAA-3′). The PCR conditions were carried out according to Cho *et al.* [6] and the PCR product was directly sequenced in both directions using both forward and reverse primers. The obtained sequences were then aligned with reference sequences available in the GenBank database using BLAST tool. The 502 bp *COX-1* gene showed 100% similarity with *T. saginata* tapeworm. Therefore, the successful amplification by PCR and the sequencing results confirmed taeniasis infection caused by *T. saginata* (Table 1).

**Table 1:** Summary of the main findings after laboratory examination of stool samples from two Palestinian patients (P-1 and P-2) admitted to health services in Ramallah and Hebron in the West Bank and diagnosed of taeniasis

Patient	Gender	Age	Parasitological findings		Molecular Diagnosis	
			Microscopic	Macroscopic	PCR^a^	Sequencing^b^
P-1	Female	33	*Taenia* ova	Proglottids	Positive	*T. saginata*
P-2	Male	22	*Taenia* ova	Proglottids/tapeworm	Positive	*T. saginata*

^a^PCR product size of 503bp [6]

^b^Sequences aligned using BLAST tool

## Discussion and conclusion

Taeniasis is considered unusual infection in Palestine since the consumption of raw meat is not among eating habits and not popular. Tapeworm infection due to *Taenia solium* is more prevalent in undeveloped communities with poor sanitation and people eat raw pork meat. This is not the case for Palestinian community, where eating pork meat is forbidden for most of the people. Fecal–oral transmission of taeniasis is considered risk factor among immigrant communities [7].

Epidemiological surveys of taeniasis infection have not been conducted in Palestine, but very few positive samples of taeniasis of unknown species reported [5], there is a growing concern that the occurrence of human taeniasis is principally due to cultural characteristics of the people and their eating habits in consumption of raw or undercooked beef. Both patients reported of consuming raw and uncooked meat.

This is the first molecular diagnostic analysis of stool samples and confirmed that both patients were infected with *T. saginata.* The use of the mitochondrial *COX-1* gene in this study allowed an accurate taxonomic identification of *T. saginata* specimens which is very important for the epidemiological characteristics of the disease in Palestine.

Further molecular analysis of the sequences from these two Palestinian patients and probably others are needed and may reveal important molecular and epidemiological information. Surveys of the prevalence of taeniasis infection and the associated factors are urgently needed in Palestine. The presence of such parasitic infection chronically can worsen the prognosis of this potentially complicated infection [8].

In conclusion, this report highlighted the existence of taeniasis infection in Palestine even with few reported numbers. This necessitates the need to conduct further research and surveillance throughout the country.

## Data Availability

Not applicable.
